# SOX2 Co-Occupies Distal Enhancer Elements with Distinct POU Factors in ESCs and NPCs to Specify Cell State

**DOI:** 10.1371/journal.pgen.1003288

**Published:** 2013-02-21

**Authors:** Michael A. Lodato, Christopher W. Ng, Joseph A. Wamstad, Albert W. Cheng, Kevin K. Thai, Ernest Fraenkel, Rudolf Jaenisch, Laurie A. Boyer

**Affiliations:** 1 Department of Biology, Massachusetts Institute of Technology, Cambridge, Massachusetts, United States of America; 2 Whitehead Institute for Biomedical Research, Cambridge, Massachusetts, United States of America; 3 Department of Biological Engineering, Massachusetts Institute of Technology, Cambridge, Massachusetts, United States of America; 4 Computational and Systems Biology Program, Massachusetts Institute of Technology, Cambridge, Massachusetts, United States of America; Stanford University School of Medicine, United States of America

## Abstract

SOX2 is a master regulator of both pluripotent embryonic stem cells (ESCs) and multipotent neural progenitor cells (NPCs); however, we currently lack a detailed understanding of how SOX2 controls these distinct stem cell populations. Here we show by genome-wide analysis that, while SOX2 bound to a distinct set of gene promoters in ESCs and NPCs, the majority of regions coincided with unique distal enhancer elements, important *cis*-acting regulators of tissue-specific gene expression programs. Notably, SOX2 bound the same consensus DNA motif in both cell types, suggesting that additional factors contribute to target specificity. We found that, similar to its association with OCT4 (*Pou5f1*) in ESCs, the related POU family member BRN2 (*Pou3f2*) co-occupied a large set of putative distal enhancers with SOX2 in NPCs. Forced expression of BRN2 in ESCs led to functional recruitment of SOX2 to a subset of NPC-specific targets and to precocious differentiation toward a neural-like state. Further analysis of the bound sequences revealed differences in the distances of SOX and POU peaks in the two cell types and identified motifs for additional transcription factors. Together, these data suggest that SOX2 controls a larger network of genes than previously anticipated through binding of distal enhancers and that transitions in POU partner factors may control tissue-specific transcriptional programs. Our findings have important implications for understanding lineage specification and somatic cell reprogramming, where SOX2, OCT4, and BRN2 have been shown to be key factors.

## Author Summary

In mammals, a few thousand transcription factors regulate the differential expression of more than 20,000 genes to specify ∼200 functionally distinct cell types during development. How this is accomplished has been a major focus of biology. Transcription factors bind non-coding DNA regulatory elements, including proximal promoters and distal enhancers, to control gene expression. Emerging evidence indicates that transcription factor binding at distal enhancers plays an important role in the establishment of tissue-specific gene expression programs during development. Further, combinatorial binding among groups of transcription factors can further increase the diversity and specificity of regulatory modules. Here, we report the genome-wide binding profile of the HMG-box containing transcription factor SOX2 in mouse embryonic stem cells (ESCs) and neural progenitor cells (NPCs), and we show that SOX2 occupied a distinct set of binding sites with POU homeodomain family members, OCT4 in ESCs and BRN2 in NPCs. Thus, transitions in SOX2-POU partners may control tissue-specific gene networks. Ultimately, a global analysis detailing the combinatorial binding of transcription factors across all tissues is critical to understand cell fate specification in the context of the complex mammalian genome.

## Introduction

Transcription factors bind DNA in a sequence-specific manner and regulate gene expression patterns in response to developmental cues. Thus, transcription factors often direct a hierarchy of events controlling cellular identity [1], [2]. The HMG box containing transcription factor SOX2 is essential for the development of the epiblast in the early mammalian embryo [3] and for the maintenance of embryonic stem cells (ESCs) *in vitro* [4]. SOX2 is also necessary for the function and maintenance of neural progenitor cells (NPCs) in the nervous system [5], [6]. Further, SOX2 functions in other adult stem cell and progenitor populations in the gastrointestinal and respiratory tract, as well as in the developing lens, inner ear, taste buds, and testes [7]–[12]. Thus, SOX2 is a critical regulator of distinct stem cell states, but how it can serve this multifunctional role is not fully understood.

In ESCs, SOX2 is a component of the core transcriptional regulatory circuitry that controls pluripotency. Together with OCT4 (*Pou5f1*) and NANOG, SOX2 binds to the proximal promoters of large cohort of genes with known roles in pluripotency (including *Oct4*, *Sox2*, and *Nanog*) as well as those that function later in development [13]–[16]. These data suggest that SOX2 regulates ESC state by actively promoting pluripotency and by marking the regulatory regions of developmental genes for future activation. Consistent with this, SOX2 can act as a pioneer factor at a subset of genes in ESCs, and can be sequentially replaced by other SOX family members during differentiation, leading to activation of genes [17], [18]. SOX2 is also a critical factor in somatic cell reprogramming, whereby adult cells are converted into a pluripotent ESC-like state by the exogenous expression of a small set of transcription factors [19]–[21], with SOX2 being at the top of a gene expression hierarchy during the late phase of reprogramming [22].

In the central nervous system (CNS), *Sox2* is required for proper NPC function during embryonic development and for maintenance of NPCs postnatally [23]–[25]. Specifically, loss of *Sox2* in the developing CNS leads to multiple brain defects, including precocious progenitor differentiation and a reduced proliferating cell population in the brain, resulting in perinatal lethality [5], [6], [26], [27]. In contrast, forced expression of *Sox2* blocks terminal differentiation of NPCs [26]–[29]. While a critical role for *Sox2* in distinct stem cell populations has been firmly established both *in vivo* and *in vitro*, the molecular mechanisms by which SOX2 regulates cell type-specific gene expression programs are not clear.

Analysis of genome-wide binding profiles indicates that SOX2 occupies the promoters of thousands of genes [17], [30], however, a direct comparison of SOX2 targets in ESCs and NPCs has not been reported. Emerging evidence indicates that transcription factors drive tissue specific gene expression programs through interactions with distal enhancer elements [31]–[33]. Recent studies have shown that histone modification patterns, specifically monomethylation of lysine 4 of histone H3 (H3K4me1) and acetylation of lysine 27 on histone H3 (H3K27ac), mark distal enhancers [34]–[37]. Using this set of histone marks, we previously identified thousands of enhancer elements in ESCs and NPCs [34]. Thus far, the binding of SOX2 at enhancers has only been clearly demonstrated at a few genes in both ESCs and NPCs. For example, SOX2 occupies the proximal and distal enhancers upstream of the *Oct4* promoter in ESCs whereas binding at an intronic enhancer (Nes30) in the *Nestin* gene was observed in NPCs [14], [38]–[40]. Thus, knowledge of SOX2-bound enhancers in these two cell types will contribute significant new insights into understanding control of cell state.

SOX family members weakly bind DNA and cannot robustly activate transcription alone, suggesting roles for additional partner factors in target selection [41]. Consistent with this, cooperation between SOX and POU transcription factor families has been highly conserved across metazoans where these factors are important regulators of developmental programs [42]. For example, SOX2 cooperates with the Class V POU family member OCT4 in ESCs to maintain pluripotency [13]–[16], however transcription factors that function with SOX2 genome-wide in NPCs are largely unknown. Thus, the identification of factors that bind to genomic sites with SOX2 will also be key to understanding how this master regulator can control distinct phenotypic outcomes.

Here, we defined the genome-wide binding patterns of SOX2 in ESCs and NPCs and show that SOX2 occupied a largely distinct set of genomic regions within promoters and distal enhancer elements in the two cell types. Similar to its cooperation with OCT4 (*Pou5f1*) in ESCs, we identified the Class III POU transcription factor BRN2 (*Pou3f2*) as a candidate SOX2 partner factor that co-bound a large fraction of distal enhancers with SOX2 in NPCs. Consistent with a functional role, forced expression of BRN2 in differentiating ESCs led to recruitment of SOX2 to a subset of NPC distal enhancers. This recruitment was associated with changes in chromatin structure, activation of neighboring genes, and ultimately precocious differentiation toward a neural-like state. Further analysis of bound sequences showed differences in the arrangement of a SOX-POU binding in ESCs and NPCs and revealed enrichment for additional transcription factor motifs. Together, these data reveal new insights into how SOX2 can function in a context-dependent manner to specify distinct stem cell states. Our work also has important implications for understanding development as well as the process of somatic cell reprogramming.

## Results

### SOX2 occupies distinct genomic regions in ESCs and NPCs

**Figure 1:**
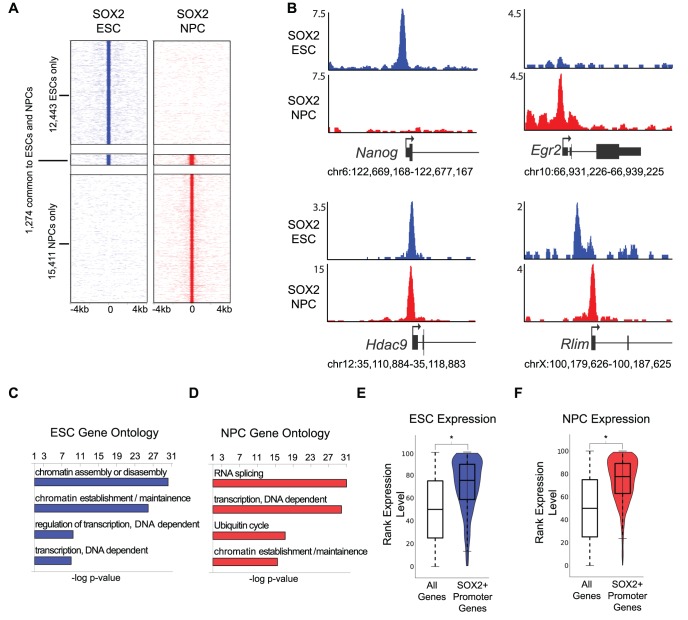
SOX2 binds promoters with cell-type-specific functions in ESCs and NPCs. (A) Heat maps of SOX2 enrichment in ESCs and NPCs centered on peaks of enrichment and extended 4 kb in each direction. (B) Gene plots showing SOX2 density at *Nanog*, *Erg2*, *Hdac9*, and *Rlim* in ESCs (blue) and in NPCs (red). y-axis corresponds to reads per million. Genomic positions reflect NCBI Mouse Genome Build 36 (mm8). (C, D) GOstat gene ontology analysis of genes linked to SOX2 bound TSSs. x-axis corresponds to negative log base ten p-value of enrichment of genes in target list compared to a whole genome background set. (E, F) Box and Violin plots representing expression data from Affymetrix arrays of genes linked to SOX2 target TSSs. y-axis corresponds to percentile expression rank, * denotes p-value<0.01, Student's T-test, two tailed.

SOX2 is a master regulator of pluripotent ESCs and multipotent NPCs, yet how the same transcription factor can specify distinct stem cell states remains an open question. We reasoned that detailed analysis of genomic binding patterns in the two cell types might reveal how SOX2 can regulate diverse gene expression programs. To this end, we differentiated ESCs toward NPCs using established protocols [43], and interrogated SOX2 binding sites by chromatin immunoprecipitation followed by massively parallel sequencing (ChIP-Seq). Analysis of SOX2 binding in genetically identical ESC and NPC lines identified 13,717 and 16,685 enriched regions, respectively (Table S1). Our results were highly consistent with prior work in ESCs [16], however we observed a lower correlation compared to published data sets in neural progenitor cells (Figure S1A and Discussion). We found that >95% of bound regions are unique to each cell type (only 1,274 of the total regions are common to both datasets) (Figure 1A and Figure S1A, S1B). Thus, we identified a union set of 29,128 enriched regions at high confidence and found that SOX2 occupied a largely non-overlapping set of genomic sites in ESCs and NPCs.

SOX2 is thought to bind to regulatory regions of genes with roles in stem cell maintenance and neural differentiation [13]–[16], however, a direct comparison of genome-wide binding in ESCs and NPCs has not been reported. Thus, we first mapped binding sites within 1 kb of a transcription start site (TSS) and found that SOX2 occupied 893 and 3,821 sites within promoters in ESCs and NPCs, respectively (Table S2). While ∼one-third (36%) of bound TSSs in ESCs were common to NPCs, SOX2 largely occupied distinct sites within promoters in the two cell types (Figure S1C). For example, SOX2 occupied the *Nanog* promoter only in ESCs, while the *Egr2 (Krox20)* promoter was bound only in NPCs, and a site within the *Hdac9* promoter was occupied in both cell types (Figure 1B). *Nanog* and *Egr2* are critical regulators of the ESC state and neural development, respectively, and *Hdac9* is a broadly expressed chromatin regulator with a known role in brain development [44]–[48]. Furthermore, we also found examples where SOX2 occupied different sites in ESCs and NPCs but within the promoters of the same gene, such as the *Rlim* promoter, which encodes a regulator of both X-inactivation and later neural patterning [49], [50] (Figure 1B). Consistent with this, while roughly one-third of TSS-associated regions overlapped in ESCs and NPCs, 58% of the genes bound by SOX2 in ESCs were also NPC targets (Figure 1B, Figure S1C and S1D). These data suggest that SOX2 can utilize different binding sites to regulate genes in a context-dependent manner.

On a global level, SOX2 bound to a set of genes that code for chromatin and transcriptional regulators in both ESCs and NPCs in accordance with previous data [13]–[16] (Figure 1C, 1D and Table S3). While many of these targets were common to both cell types, a large group of chromatin and transcriptional regulators (490) were occupied uniquely in NPCs. Moreover, SOX2 bound more promoter regions in NPCs compared to ESCs and also occupied genes with diverse functions such as RNA splicing, regulation of the ubiquitin cycle, and translation (Figure 1D). While RNA splicing is a general cellular function, alternative splicing is known to play a key role in brain development [51]. For example, in NPCs, SOX2 occupied the promoters of the alternative splicing factors PTB and nPTB, which constitute a molecular switch regulating neuronal commitment [52]. We also found that SOX2 occupied genes displayed higher expression compared to all genes (Figure 1E, 1F and Table S4) suggesting that SOX2 has a positive regulatory role at promoters in each cell type.

### SOX2 binds to distal enhancer elements in ESCs and NPCs

**Figure 2:**
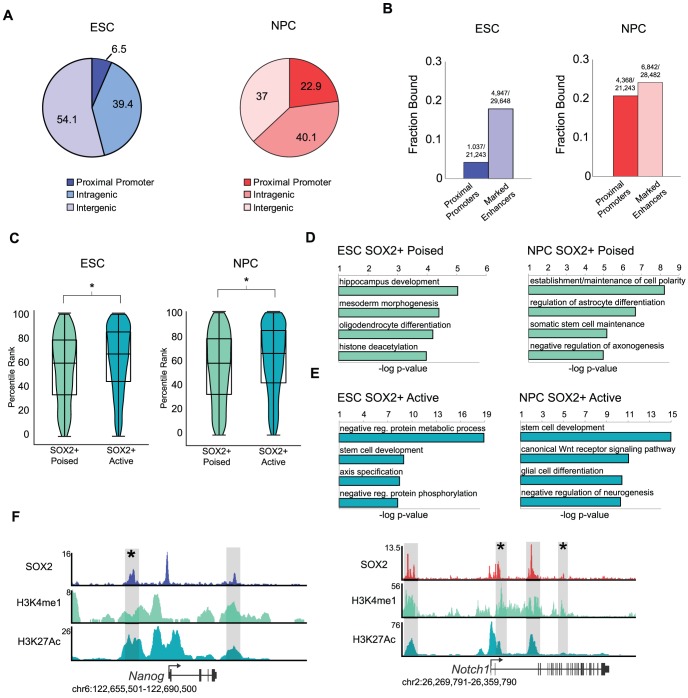
SOX2 binds distinct enhancer regions in ESCs and NPCs. (A) SOX2 peaks mapping to annotated transcriptional start sites, intragenic regions, and intergenic regions. Numbers on pie chart indicate fraction of bound regions that fall into each category. (B) Fraction of total start sites or total marked enhancers associated with SOX2 enriched regions. Numbers above bars reflect absolute numbers of bound regions and genomic features. (C) Box and Violin plots representing expression data from Affymetrix arrays of genes linked to SOX2-bound poised and active enhancers. y-axis corresponds to percentile expression rank, * denotes p-value<0.01, Student's T-test, two tailed. (D, E) Analysis of GO biological processes enriched in SOX2 bound poised and active enhancer datasets in ESCs and NPCs. x-axis reflects negative log base 10 of binomial raw p-value for enrichment versus a whole genome background. (F) Gene plots showing SOX2, H3K4me1, and H3K27Ac density at *Nanog* in ESCs and *Notch1* in NPCs. y-axis corresponds to reads per million. Genomic positions reflect NCBI Mouse Genome Build 36 (mm8). Gray boxes indicate putative enhancers occupied by SOX2. * denotes known enhancer region.

While SOX2 occupied proximal promoter regions in the two cell types, the vast majority of bound sites (>93% and >77% in ESCs and NPCs, respectively) mapped greater than 1 kb from annotated TSSs (Figure 2A). Distal enhancers are important non-coding DNA elements that control tissue specific gene expression patterns at variable distances from the promoters they regulate through binding of transcriptional and chromatin regulators [31]–[33]. We previously identified thousands of putative enhancers in ESCs and NPCs by genome-wide analysis of H3K4me1 and H3K27Ac occupancy, two histone marks known to mark distal enhancer elements [34]. SOX2 bound ∼17% (4,947) and ∼24% (6,842) of these putative enhancers in ESCs and NPCs, respectively (Figure 2B and Table S2). Currently, distal enhancers are presumed to regulate the nearest gene [34], [37], and after assigning each enhancer to the nearest upstream or downstream gene, we found that the SOX2-bound enhancers corresponded to 3,372 and 3,990 genes in ESCs and NPCs, respectively (Table S2). While these sites were largely distinct in the two cell types (Figure S2A), ∼44% of genes associated with SOX2 enhancers in ESCs also had a bound enhancer assigned to the same gene in NPCs (Figure S2B) and included many factors with specific roles in neural specification. Notably, analysis of bound enhancers revealed thousands of additional genes that may be regulated by SOX2 in both cell types which would not have been identified by analysis of only TSSs (Figure S2C, S2D). These data are consistent with the idea that, while enhancer utilization is highly cell type-specific, individual genes can be regulated by different enhancers [31], [53].

The pattern of H3K4me1 and H3K27Ac occupancy can distinguish a given enhancer as active (H3K4me1+/−; H3K27Ac+) or poised (H3K4me1+; H3K27Ac−), states which correlate with high expression of a neighboring gene or the potential of that gene to be expressed later during development, respectively [34], [37], [54], [55]. Thus, globally genes nearest active enhancers are expressed at a higher level than those linked to poised elements. By comparison of SOX2-bound regions with the set of active and poised enhancers in our previous study [34], we found that SOX2 occupied 2,100 and 4,037 poised enhancers and 2,847 and 2,805 active enhancers in ESCs and NPCs, respectively (Table S2). Consistent with the idea that enhancers regulate transcriptional output, expression of genes closest to SOX2-bound active enhancers is significantly higher than genes associated with SOX2-bound poised enhancers (Figure 2C).

To gain deeper biological insights, we used the GREAT algorithm to perform Gene Ontology (GO) analysis to determine the function of genes associated with SOX2-bound enhancers. SOX2-bound poised enhancers in ESCs were nearest genes that function in commitment to the neural lineage and morphogenesis and included *Jag1*, *Neurog3*, and *Nkx2-2*, whereas those associated with poised enhancers in NPCs included genes with roles in terminal differentiation into neurons and glia such as *Atoh1*, *Lhx8*, *Id2* and *Id4* (Figure 2D, Tables S5 and S6). Notably, SOX2 bound to active enhancers nearest genes with functions in stem cell development in both cell types. Enriched categories in ESCs also revealed genes that function in early development and axis specification whereas genes linked to active enhancers in NPCs have roles in WNT signaling and neurogenesis (Figure 2E). For example, SOX2 occupied a known enhancer in the 5′ region of the *Nanog* locus in ESCs [56] and bound to intronic enhancers in *Notch1* in NPCs [57], [58], known regulators of pluripotency and neurogenesis, respectively (Figure 2F). Thus, we identified thousands of stage-specific enhancers including many previously known enhancers in both cell types.

Despite the low overlap of SOX2-bound enhancer regions in ESCs and NPCs, genes linked to SOX2-bound poised enhancers in ESCs had functions in neural development, similar to genes linked to SOX2-bound enhancers in NPCs. Thus, we hypothesized that SOX2 might be regulating a subset of targets in both cell types by occupying distinct enhancer elements. Indeed, direct comparison of these genes revealed that ∼50% of genes (821 of 1,654) associated with SOX2-bound poised enhancers in ESCs also had a bound enhancer associated with that gene in NPCs (Figure S2E), despite the regions of SOX2 binding being largely cell-type specific. Importantly, genes where enhancers remained poised showed no significant difference in expression whereas those genes that gained active enhancers were expressed at higher levels (Figure S2F). These data are consistent with the idea that, while enhancer utilization is highly cell type-specific, individual genes can be regulated by different enhancers [31], [53]. Along those lines, using the GREAT algorithm to query the MGI gene expression database, we determined that 2,253 of the 4,037 SOX2-bound poised enhancers in NPCs were linked to genes expressed in the postnatal mouse nervous system (binomial p-value = 1.91e-35) (Table S6). Together, our data support the idea that poised enhancers can predict future developmental potential and suggest that SOX2 regulates a larger network of genes than previously anticipated by binding to distal enhancer elements.

### BRN2 co-occupies distal enhancers with SOX2 in NPCs

**Figure 3:**
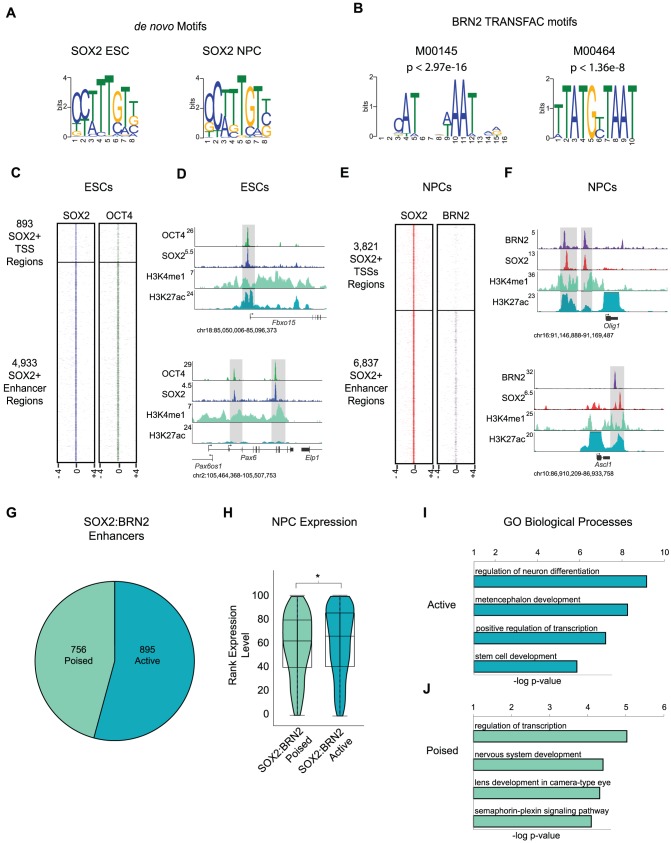
BRN2 co-occupies distal enhancers with SOX2 in NPCs. (A) *de novo* MEME motif analysis of SOX2 bound regions in ESCs and NPCs revealed canonical SOX2 motif. (B) TRANSFAC BRN2 motifs enriched in SOX2 target regions in NPCs. p-values represent significance of enrichment based on Mann-Whitney Wilcoxon ranked sum test with Benjamini-Hochberg multiple hypothesis testing correction. (C) Heat maps of SOX2 and OCT4 at SOX2 bound promoters and enhancers centered on peaks of SOX2 enrichment and extended 4 kb in each direction. (D) Gene plots showing SOX2, OCT4, H3K4me1, and H3K27Ac density at the *Fbxo15* promoter and at poised enhancers of *Pax6* in ESCs. y-axis corresponds to reads per million. Genomic positions reflect NCBI Mouse Genome Build 36 (mm8). Gray boxes indicate regions co-occupied by SOX2 and OCT4. (E) Heatmaps of SOX2 and BRN2 enrichment at SOX2-bound promoters and enhancers in NPCs centered on peaks of SOX2 enrichment and extended 4 kb in each direction. (F) Gene plots showing SOX2, BRN2, H3K4me1, and H3K27Ac density at *Olig1* and *Ascl1* loci in NPCs. y-axis corresponds to reads per million. Due to the high enrichment of H3K27Ac at active promoters, y-axis was cut off to show full dynamic range of enhancer-associated H3K27Ac density. Genomic positions reflect NCBI Mouse Genome Build 36 (mm8). Gray boxes indicate regions of SOX2-BRN2 co-occupancy. * indicates known enhancer. (G) Breakdown of number of SOX2-BRN2 target enhancers that are H3K4me1+, H3K27Ac− (poised) or H3K4me1+/−, H3K27Ac+ (active). (H) Box and Violin plots representing expression data from Affymetrix arrays of genes linked to poised and active SOX2-BRN2 target enhancers in NPCs. y-axis corresponds to percentile expression rank, * denotes p-value<0.01, Student's T-test, two tailed. (I, J) GREAT analysis of genes linked to poised and active SOX2-BRN2 target enhancers in NPCs.

SOX2 binds DNA weakly and it is insufficient to strongly activate transcription without cooperation with additional factors [59]. Consistent with this idea, we identified the canonical SOX2 motif, 5′-CTTTGTT-3′ [60]–[63] as highly enriched in ESCs and NPCs despite the difference in binding patterns (Figure 3A). Thus, we sought to identify additional factors that may function with SOX2 in ESCs and NPCs. SOX2 partners with the Class V POU-domain containing transcription factor OCT4 in ESCs to regulate a large cohort of genes important for pluripotency [13]–[16] however, partner factors in NPCs have not been clearly defined.

Interactions between SOX and POU factors are conserved in all metazoans and play key roles in embryonic development [42], thus, we hypothesized that SOX2 may also function with POU factors in NPCs. To test this, we interrogated a 100 bp window surrounding peaks of SOX2 enrichment in NPCs and determined enrichment for all known vertebrate transcription factor-binding motifs in the TRANSFAC database. Notably, we identified several enriched motifs, including two highly similar motifs recognized by the Class III POU factor BRN2 (*Pou3f2*) (Figure 3B and Table S7). BRN2 was of particular interest for several reasons. First, our transcriptome analysis showed that *Brn2* is highly expressed in NPCs, but not in ESCs (Table S7). Moreover, *Brn2* and *Sox2* are both expressed in neurogenic regions of the brain and SOX2 and BRN2 are known to co-occupy a small number of loci in this tissue [38], [64], [65]. Like *Sox2*, *Brn2* loss-of-function causes pleiotropic defects and NPC impairment [66]–[69]. Furthermore, *Sox2*, *Brn2*, and the forkhead transcription factor *Foxg1* are sufficient to reprogram fibroblasts toward a multipotent NPC-like state [70]. These data suggest that transitions in POU partner factors of SOX2 may control cell identity in distinct stem cell populations.

Although neurogenesis and maintenance of cell identity in the brain require BRN2, its target genes in NPCs were not known. To address this, we performed ChIP-Seq and identified 6,574 BRN2 occupied regions in NPCs (Table S1). Similar to SOX2-bound regions, more BRN2-bound regions mapped to previously identified distal enhancers [34] than to promoter regions (Figure S3A). Motif analysis revealed enrichment for a canonical Octamer (OCT) motif (5′-ATGCATAT -3′) [71], [72] within BRN2 bound sites validating the high quality of our data set (Figure S3B).

We next examined the overlap between SOX2 and the two POU factors (BRN2 and our previously published OCT4-ESC dataset [16], Table S1) in ESCs and NPCs. Regions occupied by OCT4 and BRN2 showed little overlap (Figure S3C), indicating that these factors occupied cell-type-specific targets. Our data confirmed that SOX2 and OCT4 co-occupied many genomic sites in ESCs [13]–[16] (Figure 3C and Figure S3D-S3G). For example, SOX2 and OCT4 co-bound the promoter of *Fbxo15* and to two putative enhancers of *Pax6* that have been previously identified based on evolutionary sequence conservation and histone modification patterns [73] (Figure 3D). Notably, whereas BRN2 was absent from most SOX2-bound promoters in NPCs (Figure 3E), BRN2 occupied a subset of distal enhancers and bound many of these sites with SOX2, including known SOX2-BRN2 targets such as enhancers of *Sox2* and *Nestin* [38], [65] (Figure S3H-S3K). For example, SOX2 and BRN2 co-occupied putative 3′ enhancer regions of *Olig1* [74], and a known regulatory region 3′ of the *Ascl1 (Mash1)* locus [75] (Figure 3F). Together, these data suggest that SOX2 functions with BRN2 at a subset of distal enhancers to regulate target genes in NPCs.

Whereas SOX2-OCT4 bound enhancers associated with genes that have roles in pluripotency and lineage commitment, SOX2-BRN2 enhancers neighbored genes that function in NPC identity. Overall, SOX2 and BRN2 occupied 756 poised and 895 active enhancers in NPCs (Figure 3G). SOX2-BRN2 bound active enhancers correlated with genes that were expressed at higher levels than those associated with poised enhancers (Figure 3H). Further analysis revealed genes linked to active enhancers included transcription factors that play roles in neural development such as *Notch1, Rfx4* and *Sox2* itself (Figure 3I and Table S8). Interestingly, genes linked to the co-bound poised enhancers in NPCs included regulators of later stages of neuronal developmental such as the pro-neural transcription factor *Atoh1* [76], [77] and *Dab1*, a critical regulator of neuroblast migration [78] (Figure 3J and Table S8). Notably, ∼24% of genes associated with SOX2-OCT4 poised enhancers in ESCs overlapped with genes associated with SOX2-BRN2 bound enhancers in NPCs that included known regulators of neural development such as *Atoh1* and *Ncam1*, despite differences in the bound regions. Thus, SOX2-POU partnerships may control neural development by differentially targeting specific subsets of enhancers in pluripotent ESCs and multipotent NPCs, in order to establish the development potential of this tissue from very early stages of embryogenesis.

### BRN2 expression in ESCs promotes neural differentiation

**Figure 4:**
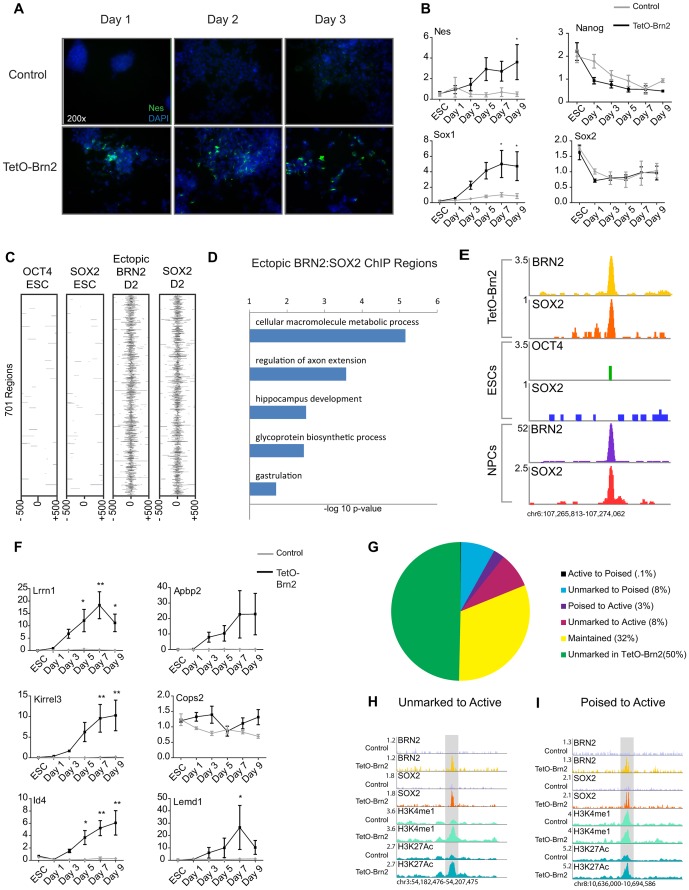
Brn2 biases ES cells towards neural differentiation. (A) Staining with DAPI (blue) and immunocytochemistry of NESTIN (green) in ESCs induced to differentiate in adherent cultures with or without ectopic *Brn2*. (B) qRT-PCR of the indicated genes in ESCs with (black lines) and without (gray lines) ectopic *Brn2* expression through differentiation. y-axis represents relative expression normalized to *Gapdh* in 3 biological replicates, measured in triplicate. ESC time point is ESCs without doxycycline, and d1–d9 time points represent time in differentiation medium. * denotes p-value<0.05, ** denotes p-value<0.01 ANOVA with Bonferroni correction (C) Heatmap of OCT4 and SOX2 enrichment in ESCs and ectopic BRN2 and SOX2 in TetO-Brn2 cells of 701 genomic regions occupied by only ectopic BRN2 and SOX2. (D) GREAT GO biological processes enriched in 701 regions in (C). x-axis reflects negative log base 10 of raw binomial p-value for enrichment versus a whole genome background. (E) Gene plots depicting peaks of enrichment in indicated datasets at a locus distal to *Lrrn1*. y-axis corresponds to reads per million. Genomic positions reflect NCBI Mouse Genome Build 36 (mm8). (F) qRT-PCR of genes associated with SOX2-BRN2 binding in TetO-Brn2 cells and NPCs, in ESCs with (black lines) and without (gray lines) ectopic *Brn2* expression through differentiation. y-axis represents relative expression normalized to *Gapdh* in 3 biological replicates, measured in triplicate. ESC time point is ESCs without doxycycline, and d1–d9 time points represent time in differentiation medium. * denotes p-value<0.05, ** denotes p-value<0.01 ANOVA with Bonferroni correction. (G) Pie chart reflecting overlap between SOX2-BRN2 regions and enhancer chromatin marks in TetO-Brn2 cells. Percentages in legend reflect fraction of 1,533 SOX2-BRN2 regions in each category. (H) Example region that was occupied by ectopic BRN2 in TetO-Brn2 cells, leading to recruitment of endogenous SOX2 and the deposition of H3K4me1 and H3K27Ac. y-axis corresponds to reads per million. Genomic positions reflect NCBI Mouse Genome Build 36 (mm8). Gray box indicates region of SOX2-BRN2 co-occupancy in TetO-Brn2 cells which was not occupied by SOX2 in control cells. (I) Example poised enhancer occupied by ectopic BRN2 in induced cells, leading to recruitment of endogenous SOX2, and deposition of H3K27Ac. y-axis corresponds to reads per million. Genomic positions reflect NCBI Mouse Genome Build 36 (mm8). Gray box indicates region of SOX2-BRN2 co-occupancy in TetO-Brn2 cells which was not occupied by SOX2 in control cells.

The significant overlap between BRN2 and SOX2 in NPCs predicts that BRN2 is also an important driver of neural commitment. To test this idea, we generated ESC lines that harbored a drug-inducible *Brn2* transgene (TetO-Brn2) and assayed the potential of these cells to differentiate toward the neural lineage (Figure S4A–S4C). Upon *Brn2* induction, ESCs showed distinct morphological changes from round cells that grew in colonies to polarized, Nestin-positive cells at day 1 of differentiation compared to control cells (Figure 4A and Figure S4D). Consistent with these changes, neural lineage genes such as *Nestin* and *Sox1* showed higher expression in ESCs upon *Brn2* expression (Figure 4B). Notably, *Brn2* induction led to changes in gene expression and cell fate in the absence of additional growth factors whereas control cells did not show significant differences under these conditions. Thus, forced expression of *Brn2* can promote differentiation of ESCs toward a neural-like fate.

Our data suggested that POU factor expression may be a key determinant of cell-type-specific SOX2 target selection, so we hypothesized that ectopic BRN2 might be sufficient to recruit endogenous SOX2 to genomic regions *de novo.* To test this, we collected TetO-Brn2 cells two days after induction (Figure S4D) and performed ChIP-Seq. We identified 12,362 and 8,401 regions occupied by SOX2 and BRN2 in these cells, respectively (Table S1). Similar to SOX2 and BRN2 in NPCs, these factors occupied more distal sites than promoters (Figure S4E). Strikingly, ∼18% (1,034 regions) occupied by BRN2 in the induced ESCs were also bound by BRN2 in NPCs, indicating that ectopic BRN2 retained some of its NPC target specificity. These regions were distal to loci encoding neurodevelopmental regulators such as Ephrin Receptors (*Epha3*, *Epha4*, *Epha5*, *Epha7*) and transcription factors such as *Id2* and *Id4* (Table S9).

Importantly, we defined 1,533 regions co-occupied by BRN2 and SOX2 in these cells. Comparison of these regions to SOX2 and OCT4 targets in ESCs and SOX2 in control cells at day 2 (Table S1) revealed 701 (46%) of these sites were bound uniquely by SOX2-BRN2 in the induced cells. These data suggested that BRN2 was necessary for SOX2 binding at these sites (Figure 4C and Figure S4F, S4G). Analysis of enriched GO categories showed that genes closest to these novel targets had roles in the development and function of the nervous system (Figure 4D and Table S9). Notably, 21% of these novel sites (144 regions) were also bound by SOX2 and/or BRN2 in NPCs, including enhancers linked to genes with demonstrated roles in neural development such as *Lrrn1* and *Abpa2 (X11l)* [79]–[81] (Figure 4E). Expression analysis by qRT-PCR of a subset of these TetO-Brn2/NPC, SOX2-BRN2 genes, including *Lrrn1*, *Abpa2*, *Kirrel3, Cops2, Id4*, and *Lemd1*, revealed that some were significantly induced in TetO-Brn2 cells compared to controls (Figure 4F). Thus, ectopic BRN2 was sufficient to recruit SOX2 to NPC-specific sites and to induce the expression of nearby genes, indicating that POU-factor partners are sufficient to functionally recruit SOX2 to a subset of cell-type-specific target loci.

Given that SOX2-BRN2 binding in NPCs correlated with cell-type-specific distal enhancers, we hypothesize that SOX2-BRN2 might play a role in regulating the state of these elements. Thus, we next examined the distribution of the enhancer chromatin marks, H3K4me1 and H3K27Ac, in TetO-Brn2 and control cells at day 2 in order to determine whether the ectopic binding of these factors could alter local chromatin structure (Table S1). We found that 777 of the 1,533 co-bound sites (∼51%) were coincident with H3K4me1 and/or H3K27Ac regions in TetO-Brn2 cells and 488 of these regions (∼32%) displayed similar patterns in both induced and control cells (Figure 4G). Interestingly, 165 of the co-occupied regions (∼11%) gained H3K27Ac upon *Brn2* induction, and were closest to genes involved in neural development such as *Atoh1*, *NeuroD1*, and *Tcf7l (Tcf3)*. This included 125 regions (∼8%) that were unmarked (i.e. lacked H3K4me1 or H3K27Ac) in control cells (Figure 4H) and 40 regions (∼3%) that transitioned from a poised state to active (Figure 4I). Thus, ectopic BRN2-SOX2 binding was sufficient to activate both poised and unmarked enhancers, supporting a role for these factors in controlling global gene expression networks by regulating the activity of *cis*-regulatory elements. Collectively, these data support a role for distinct POU factors in SOX2 binding site selection and gene regulation, and suggests a model by which BRN2 functions with SOX2 to mediate developmental transitions in the neural lineage.

### Binding configurations of SOX2 and POU factors at genomic targets in ESCs and NPCs

**Figure 5:**
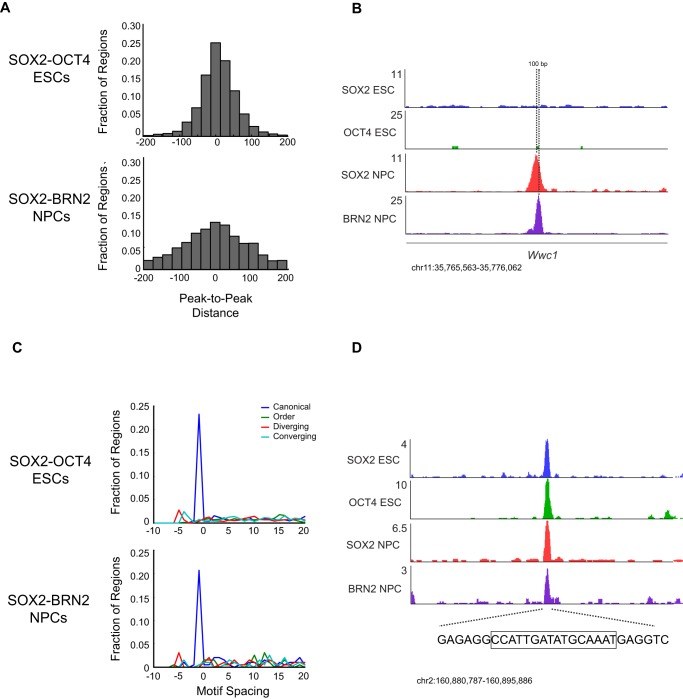
Motif configuration affects binding by SOX2 and cell-type-specific POUs. (A) Frequency distribution of distances in 25 base pair bins between peaks of OCT4 (top) and BRN2 (bottom) from SOX2 bound peaks. (B) Gene plots at the *Wwc1* locus. Direction of transcription (5′-3′) is left to right. Hashed line represents position of peaks of SOX2 and BRN2 enrichment separated by 100 bp. y-axis corresponds to reads per million. Genomic positions reflect NCBI Mouse Genome Build 36 (mm8). (C) Distribution of orientation of and distance in base pairs between of SOX and POU motifs within SOX2-OCT4 (top), SOX2-BRN2 (bottom) bound regions. Negative values on x-axis reflect instances where SOX and OCT TRANSFAC motifs overlap. y-axis reflects fraction of occurrences of indicated spacing and orientation of all bound regions which contain a SOX and OCT motif. (D) Gene plots 3′ of the *Chd6* locus, which contains a SOX-OCT motif in the canonical orientation with a −1 bp spacer. Hashed line represents sequence under the peaks of enrichment, and boxed sequence represents the canonical SOX-OCT motif with a −1 bp spacer at this locus. y-axis corresponds to reads per million. Genomic positions reflect NCBI Mouse Genome Build 36 (mm8).

Given that most SOX and POU family members bind highly similar motifs, we hypothesized that distinct motif configurations may explain, in part, the diverse binding patterns in ESCs and NPCs. For example, SOX2 and OCT4 bind DNA in distinct conformations depending on the arrangement of binding sites 82–86 and these configurations have consequences on factor binding and transcriptional outcome [38], [82], [86], [87]. We found that SOX2 frequently occupied sites within 25-bp of OCT4 (∼25%), and that relatively few sites were greater than 100–200 bp from OCT4 (∼8%) (Figure 5A). In contrast, while a significant fraction of regions showed SOX2 and BRN2 bound within 25-bp in NPCs (∼12%), a larger fraction (∼33%) occurred at distances of 100–200 bp. For example, an intragenic region of the *Wwc1* locus was bound by SOX2 and BRN2 in NPCs and peaks of enrichment were 100 bp apart (Figure 5B), while in ESCs neither SOX2 nor OCT4 recognized this element. These data indicate that while SOX2 and POU factors occupied similar motifs in ESCs and NPCs, these factors bound to different arrangements of these motifs in a cell type-specific manner.

Many known SOX2-OCT4 target sites comprise a composite SOX-Octamer (OCT) motif, consisting of a 5′-SOX motif followed by a 3′ OCT site [15], [88], [89]. Therefore, we further analyzed the configuration of the SOX2 and OCT motifs by directly inspecting the sequence within the co-occupied regions. SOX-OCT composite motifs can exist in several configurations that were previously termed “canonical”, “order”, “diverging”, and “converging” [86] (Figure 5C). Interestingly, these configurations were shown to determine which combinations of SOX and POU factors could co-occupy a given site. Surprisingly, we observed that the canonical orientation with a 1 bp overlap between the native TRANSFAC motifs was the most highly represented configuration in both SOX2-OCT4 co-bound regions in ESCs (∼23% of motif pairs) and SOX2 and BRN2 co-bound regions in NPCs (∼21% of motif pairs) (Figure 5C). For example, at a locus on chromosome 2 distal to *Chd6*, SOX2-OCT4 occupied a canonical motif with a 1 bp overlap in ESCs, and SOX2-BRN2 occupied the same site in NPCs (Figure 5D). Thus, SOX2-OCT4 and SOX2-BRN2 prefer the same composite SOX-OCT motif at genomic targets in ESCs and NPCs.

### Distinct SOX-POU sites harbor recognition motifs for other transcription factor families

**Figure 6:**
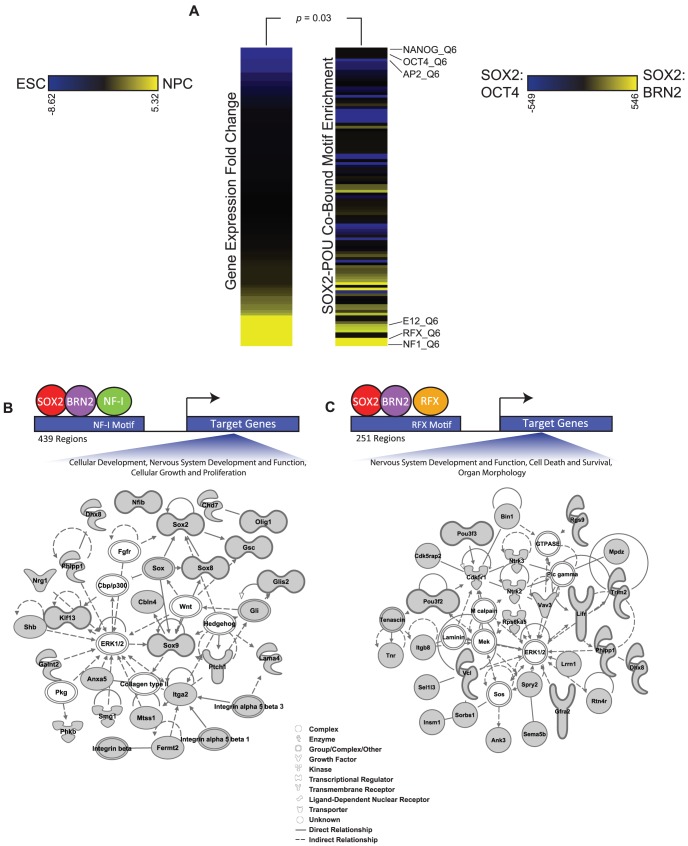
Additional transcription factor motifs are enriched in SOX2-POU-bound regions. (A) Heatmaps display the relationship between expression changes in transcription factors and the enrichment of their motifs in SOX2-BRN2 bound regions compared to SOX2-OCT4-bound regions. The full set of TRANSFAC motifs were ranked by statistical significance of enrichment in SOX2-BRN2-bound regions and in SOX2-OCT4-bound regions using the Mann-Whitney Wilcoxon test. The heat map on the right displays the change in rank of 108 TRANSFAC motifs between the two datasets. Only motifs that were ranked in the top 200 in either dataset are shown. The heat map on the left shows the fold change in gene expression between ESCs and NPCs of a transcription factor that recognizes the corresponding motif on the right. Scale bars: Left, fold gene expression change of transcription factors between ESCs (blue) and NPCs (yellow); Right, change in rank of TRANSFAC motifs between SOX2-OCT4 bound regions (blue) and SOX2-BRN2 bound regions (yellow). p-value reflects correlation of motif enrichment and gene expression of transcription factors which can recognize the motifs by a Monte-Carlo analysis. (B, C) Ingenuity Pathway Analysis to visualize the functional interconnection among genes associated with SOX2-BRN2 regions that also contain either an NF-I or RFX motif.

Combinatorial interactions among transcription factors are important for driving specific transcriptional responses [90]–[93]. In ESCs, SOX2 and OCT4 are known to co-occupy genomic sites with a cohort of other transcription factors, including NANOG, SALL4, and TCF7L1 [13]–[16], [94]–[96]. Thus, we sought to identify additional transcription factors that may interact with SOX2 and BRN2 in NPCs. To this end, we analyzed SOX2-BRN2 bound regions for enrichment of known transcription factor motifs (Table S10). To discover factors that may function specifically with SOX2 and BRN2 in NPCs, we contrasted these motifs with those that were enriched in SOX2-OCT4 co-bound regions. Notably, the enriched motifs in SOX2-BRN2 regions corresponded to transcription factors that were highly expressed in NPCs relative to ESCs (Monte Carlo analysis, p-value = 0.03, Materials and Methods) (Figure 6A). For example, NF-I motifs were highly enriched in SOX2-BRN2 regions in NPCs and family members such as *NF-Ia*, *NF-Ib*, and *NF-Ix* were expressed at significantly higher level in NPCs than ESCs (Table S10). NF-I factors have known roles in central nervous system formation and in NPC function [97]. Motifs associated with the RFX family were also enriched in SOX2-BRN2 regions (Table S10). RFX family members play essential roles in early nervous system patterning [98], [99]. While *Rfx3*, *Rfx4*, and *Rfx7* were expressed at significantly higher levels in NPCs, *Rfx2* expression was higher in ESCs (Table S10). Interestingly, a recent proteomic study identified RFX3 and NF-IB as putative SOX2 interaction partners in NPCs [30]. Thus, our analysis has identified additional transcription factors that may regulate specialized gene networks with SOX2 and POU factors in ESCs and NPCs.

We identified 439 SOX2-BRN2-NF-I-motif and 251 SOX2-BRN2-RFX-motif regions in NPCs (see Materials and Methods). Further analysis showed that SOX2-BRN2 regions containing NF-I or RFX motifs were largely exclusive (only 34 common regions) suggesting that SOX2-BRN2 sites could be further classified by interactions with specific sets of transcription factors. Consistent with this observation, SOX2-BRN2 regions containing an NF-I motif were linked to genes with functions in nervous system development and cell growth, including *Sox2* and *NF-Ib* themselves as well as *Olig1* and Integrin genes (Figure 6B). In contrast, SOX2-BRN2-RFX-motif regions were linked to a largely distinct set of regulators of neural development including regulators of neuronal apoptosis such as *Ntrk2 (TrkB)* [100], *Ntrk3 (TrkC)* [101], and *Cdk5r1 (p35)* [102], [103], an important process regulating the development of the CNS (Figure 6C). Interestingly, conditional ablation of Sox2 in NPCs is associated with increased apoptosis in the developing brain [6]. Thus, RFX and NF-I family members represent additional candidate partner factors in NPCs that may further contribute to specific regulation at SOX2-BRN2 target genes. Collectively, our work reveals a detailed picture of how SOX2 coordinates gene expression programs during lineage commitment and provides novel insights into the key principles that underpin regulation of diverse stem cell states.

## Discussion

The HMG-box transcription factor SOX2 has critical roles in the function of multiple stem cell types including pluripotent embryonic stem cells (ESCs) and multipotent neural progenitor cells (NPCs). How this master regulator can control diverse transcriptional programs has remained an important and unresolved question in the field. While SOX2 occupied many promoters in both cell types, the major class of genomic elements occupied by SOX2 in ESCs and NPCs were distal enhancers (Figure 1 and Figure 2). While our data displayed high concordance among replicates and with published data sets in ESCs, SOX2 binding in NPCs was less correlated with prior data [17], [30] (Figure S1). This is likely due to the different protocols used to derive and culture NPCs. NPCs with similar developmental potential but distinct molecular profiles exist throughout development [24], and these populations respond differently to external signaling cues present in culture media [104]–[106]. Thus, it is perhaps not surprising that SOX2 binding is more variable in NPCs relative to ESCs.

We derived NPCs directly from genetically identical ESCs allowing us to directly analyze SOX2 binding as these cells transition between states. Several criteria support the high quality of our data. First, we identified many known SOX2 binding sites including promoters and enhancers in both ESCs and NPCs. Second, while many binding sites were distinct, we identified a canonical SOX2 motif as highly enriched in both cell types. Third, SOX2 overlapped significantly with POU partner factors in ESCs and NPCs consistent with the expectation that these transcription factor families function together to regulate developmental progression. In addition, we identified a SOX-OCT composite motif as enriched in these co-bound sites.

SOX2 occupied largely exclusive sites in ESCs and NPCs, despite using the same DNA motif to recognize these genomic targets. Moreover, SOX2 occupied distinct regions in the same promoter and distinct enhancers associated with the same gene. These data indicated that additional factors dictated SOX2 binding site specificity. While SOX2 co-occupied many binding sites with OCT4 in ESCs, partner factors in NPCs have not been well defined. We found the recognition motif for the Class III POU factor BRN2 was enriched in SOX2 bound regions in NPCs. The evolutionary conservation of the SOX-POU interaction, the co-expression of *Sox2* and *Brn2* in neurogenic regions of the brain, and the neurodevelopmental defects associated with *Brn2* loss-of-function suggested that SOX2 and BRN2 together regulate a subset of genes important for neural fate. Consistent with this, we defined a large group of enhancer elements co-bound by SOX2 and BRN2 in NPCs. We identified known functional enhancers bound by SOX2 and BRN2 in NPCs, such as the *Nes30* enhancer of the *Nestin* locus [38], [40] and the 3′ enhancer of the *Sox2* locus, *SRR2* [5], and extended this list to include hundreds of additional neural-specific enhancers.

Consistent with a positive role in regulating neural cell state, forced expression of *Brn2* led to up-regulation of neural markers and to differentiation toward the neural lineage. Our work is in agreement with several studies that have implicated *Brn2* as an early marker of neural commitment [40], [107], [108]. Notably, ectopic BRN2 was sufficient to recruit SOX2 to hundreds of novel sites in differentiating ESCs that corresponded to a subset of enhancers also bound in NPCs. The recruitment of SOX2 by BRN2 to specific loci was sufficient to induce expression of nearby genes and to alter chromatin state in some cases. These data are in agreement with the notion that SOX proteins require partner factors to tightly bind to genomic targets and modulate transcriptional outcomes [59]. Interestingly, ectopic expression of OCT4 alone in NPCs was sufficient to reprogram cells into induced pluripotent stem cells, presumably by partnering with endogenous SOX2 [109], consistent with the idea that POU factors can recruit SOX2 to specific targets. Furthermore, ectopic expression of *Sox2*, *Brn2*, and the forkhead factor *Foxg1* can transdifferentiate fibroblasts to NPC-like cells [70]. Taken together, these data may facilitate efforts to define the minimal set of genes needed to promote the transition from undifferentiated cells to the neural lineage. Thus, our results implicate BRN2 as a SOX2 partner factor and suggest that together these factors are important for neural specification and NPC function.

While the motifs occupied by these factors were highly similar, the arrangement of SOX and OCT motifs in SOX2-POU target sites displayed differences in ESCs and NPCs. Regulation of SOX-POU target genes appears to depend not only on the presence of a SOX and an OCT motif in close proximity to each other, but also on other DNA sequence determinants, including the spacing and orientation of these motifs with respect to each other [82], [84], [87], [110]–[113]. However, these observations related to only a few genes and had not been extended genome-wide. While we found that SOX2-OCT4 and SOX2-BRN2 preferred similar composite motifs when they were bound in close proximity to each other, examination of co-bound regions found that peaks of SOX2 and BRN2 in NPCs were often spaced farther apart than peaks of SOX2 and OCT4 in ESCs. Thus, allosteric interactions between transactivation domains of SOX and POU factors may be key in stabilizing ternary complexes and in setting the stage for additional interactions that determine binding specificity and transcriptional output at target genes [84]–[87], [114], [115].

Combinatorial interactions among transcription factors allow cells to respond to environmental and developmental cues in a tissue-specific manner. A classical example involves the regulation of interferon-β expression through cooperative binding of transcription factors and chromatin proteins to an enhancer, collectively known as the interferon-β enhancesome [116]. In ESCs, SOX2 and OCT4 are known to physically interact with other transcription factors at many loci, including enhancers [14], [94], [117]–[120], suggesting that SOX2-POU factors may also nucleate specific enhancesomes. We identified a set of candidate factors that may interact with SOX2-BRN2 that included RFX and NF-I family members. NF-I factors are expressed in NPCs *in vivo* and their loss in development leads to defects in central nervous system formation and specifically NPC dysfunction [97], [121]–[124]. RFX family members also play essential roles in proper brain development [99], [125]. For example, RFX4 regulates Sonic Hedgehog (SHH) signaling in the developing nervous system and loss of function resulted in pleiotropic brain defects linked to SHH signaling [99], [125]. Defects associated with conditional ablation of *Sox2* in the brain were also shown to be partially mediated by aberrant SHH signaling [6]. Additional studies revealed that SOX2 co-localized with the ATP-dependent histone remodeler CHD7 in NPCs [30]. Thus, interactions with chromatin modifiers or other epigenetic regulators may also be critical for binding site selection and establishment of NPC-specific gene expression programs in response to particular signals.

Recent data showed that SOX2 functions as a pioneer factor in ESCs by marking a subset of genes for activation by other SOX family members, namely SOX4 in the B-cell lineage, SOX3 in NPCs and SOX11 in immature neurons [17], [18]. Interestingly, the POU factor *Brn5*, like *Sox11*, is expressed in differentiated cell types in the CNS and thought to play a role in regulating cell state [126]–[130], thus elucidation of BRN5 targets in these cells may reveal another layer of SOX-POU regulation of neurogenesis. Taken together, these data suggest that transitions in SOX-POU partners regulate the earliest stages of development through terminal differentiation. Ultimately, characterization of combinatorial interactions among transcription factors and chromatin regulators at distal enhancers will be central to understanding the complex mechanisms that control cell state throughout development.

## Materials and Methods

### Data deposition

ChIP-Seq and Affymetrix microarray data are deposited on GEO database under the accession numbers GSE38850 and GSE35496.

### Cell growth and culture conditions

C57/BL6-129JAE (V6.5) mouse embryonic stem cells were cultured in as described [34]. Neural progenitors were derived via *in vitro* differentiation from V6.5 ESCs as described [43] and cultured on 15 µg/ml polyornithine and 1 µg/ml laminin in N3 medium, supplemented with 5 ng/ml bFGF, 20 ng/ml EGF, and 1 µg/ml laminin. In the presence of growth factors the vast majority of these cells can be labeled homogenously with antibodies against NESTIN, SOX2, and PAX6. Upon growth factor withdrawal, the cells differentiate into TUJ1-positive neurons.

### Chromatin immunoprecipitation (ChIP) and library preparation

ChIP in NPCs was performed as described previously [131]. Briefly, approximately 5×10^8^ cells were cross-linked and chromatin fractions were sheared by sonication. ChIP-enriched and input DNA were purified and genomic libraries were prepared using the ChIP-Seq Sample Prep Kit (Illumina 1003473) according to the manufacturers protocol (Illumina 11257047) for selecting library fragments between 200 and 350 bp. Samples were run using the GA2X genome sequencer (SCS v2.6, pipeline 1.5).

For ChIP in ESCs, and in TetO-Brn2 cells and control cells were cross-linked and harvested as above. Approximately 5×10^7^ formaldhyde-crosslinked cells were lysed and as above on an IP-Star (Diagenode). Chromatin was sonicated on the Bioruptor (Diagenode) to an average size of 0.2–1 kb. ChIP was performed on chromatin from approximately 5 million cells with 3 µg of antibody (above) using the IP-Star Automated System (Diagenode) and 2.5% of chromatin was used for each whole cell extract (WCE). Following reversal of crosslinks, sample and WCE DNA was purified. ChIP and WCE DNA was dissolved in water and barcoded genomic libraries were prepared using the TruSeq DNA Sample Prep Kit (Illumina) and multiplexed on the HiSeq 2000 (Illumina).

### ChIP antibodies

Antibodies used in ChIP experiments are as follows: SOX2 (R and D Systems AF2018 goat polyclonal); BRN2 (Santa Cruz Biotechnology sc-6029 goat polyclonal); H3 (rabbit polyclonal Abcam ab1791) H3K4me1 (rabbit polyclonal Abcam ab8895); H3K27Ac (rabbit polyclonal Abcam ab4729).

### ChIP–seq analysis pipeline

Images acquired from the Illumina/Solexa sequencer were processed using the bundled Solexa image extraction pipeline. Sequences were aligned using Bowtie (http://bowtie-bio.sourceforge.net/index.shtml) using murine genome NCBI Build 36 (UCSC mm8) as the reference genome with default settings for mismatch tolerance and non-unique mapping events. Mapped reads were analyzed as described [16]. Briefly, sequence reads were extended 200 bp for transcription factors and 400 bp for histone modifications and allocated in 25 bp bins. Statistically significant enriched bins were identified using a Poissonian background model, with a p-value threshold of 10^−8^ to minimize false positives. We then used an empirical background model (whole cell extracts (WCE) for transcription factors or pan-histone histone H3 ChIP-Seq (H3) for chromatin marks) that requires bins to be enriched relative to background to eliminate non-random enrichment. Replicate datasets were combined and analyzed in one batch. Previously published datasets for enhancer associated histone marks were analyzed as described [34], [132]. SOX2, BRN2, and OCT4 enriched regions within 1 kb of a TSS were assigned to the associated gene, while bound enhancers were identified as regions that overlap H4K4me1 and/or H3K27Ac regions that are >1 kb from a TSS [34] and were assigned to the nearest gene using the GREAT algorithm for gene ontology studies and using the Galaxy web tool for all other analyses.

### ChIP–enrichment plots

ChIP–seq plots for individual genes were generated using the UCSC Genome Browser (http://genome.ucsc.edu/cgi-bin/hgGateway).. wig files were generated from ChIP-Seq reads and density was normalized to reads-per-million. Published datasets were used to correlate SOX2 bound regions to histone modification patterns for enhancer analysis [34], [132].

### Comparison of ChIP–seq datasets

We used a 1-bp minimum cutoff for the overlap between regions to define common genomic targets, as described throughout the manuscript to define co-bound SOX2-POU sites and sites occupied by SOX2 or POU factors across cell types. Correlation of ChIP-Seq datasets in figure S1 was performed using a similarity metric based on a correlation coefficient [133]. This analysis generates a correlation coefficient between zero and one reflecting the similarity of genomic regions occupied in two datasets.

### RNA isolation and microarray analysis

RNA was isolated using Trizol reagent (Invitrogen) according to the manufacturer's protocol and DNAse treated using the DNA-Free RNA kit (Zymo Research R1028). Samples were then prepared for Affymetrix GeneChip Expression Array analysis. 5 µg total RNA was used to prepare biotinylated cRNA according to the manufacturer's protocol (Affymetrix One Cycle cDNA Synthesis Kit). Samples were prepared for hybridization, hybridized to arrays, and washed according the Affymetrix hybridization manual using the Affymetrix GeneChip Hybridization, Wash and Stain Kit. GeneChip arrays (Mouse 430) were hybridized in a GeneChip Hybridization Oven at 45°C for 16 hours at 60 RPM. Arrays were scanned on a GeneChip Scanner 3000 and images were extracted and analyzed using GeneChip Operating Software v1.4.

To define expression levels of genes linked to bound promoters and enhancers, and to define fold change of expression levels of transcription factors linked to enriched TRANSFAC motifs between ESCs and NPCs, biological replicates were analyzed using the Affymetrix GCOS program and the mean intensity for each probe across three arrays was calculated. Maximum probe mean values for each gene were taken as gene expression levels. Box and Violin plots were constructed depicting median values as the center line, and bottom and top of the box representing the 25^th^ and 75^th^ percentiles, respectively. Whiskers depict+1.5*IQR (interquartile range) for top, −1.5*IQR for the bottom. To define differentially expressed genes, array data was RMA normalized using updated annotation from the BrainCDF the site and remapped from Ensembl Gene ID to Gene Name using Biomart table. For finding differentially expressed (DE) genes, the biological replicates were subjected to moderated welch test (MWT). Genes were called differentially expressed if the MWT FDR<0.05 and the fold change of the mean of the replicates was more than 1.5 fold up or down.

### Gene ontology

Gene ontology analysis was performed using GOSTAT (http://gostat.wehi.edu.au/cgi-bin/goStat.pl) for genes linked to SOX2 bound promoters or the GREAT algorithm [134] (http://great.stanford.edu/) for regions associated with SOX2 bound enhancers. GOSTAT was performed using the mgi (mouse) GO annotation database for promoter-associated regions. Since GREAT analysis requires inputs in the mm9 genome build, lift-over of mm8 called regions was performed using the Galaxy web tool prior to input into GREAT (*main.g2.bx.psu.edu/).* In general, terminal “GO Biological Process” terms were presented in figures to maximize the specificity of the information presented. In some cases terminal terms contained few genes and were thus misleading, so more informative parent terms encompassing less specific but more relevant descriptions of biological processes are presented.

### *De novo* motif enrichment analysis

*MEME* (meme.sdsc.edu/) [135] was used to find DNA sequences enriched in SOX2-, OCT4-, and BRN2-bound regions in ESCs and NPCs. Plus/minus 75 base pairs surrounding a subset of the highest peaks of enrichment for each factor (minimum peak height 100 for SOX2, 148 for OCT4, or 225 for BRN2) were input into MEME and motif logos were generated from obtained position weight matrices.

### Generation of inducible *Brn2* ESC lines

*Brn2* inducible ESCs were generated using the “flp-in” system described previously [136]. Briefly, a single copy of a tetracycline inducible mouse *Brn2* cDNA were flipped into the *Col1a1* locus of KH2 ESCs harboring an M2-rtTA gene in the *Rosa26* locus.

### ESC differentiation

Inducible *Brn2* and control (KH2) ESCs were passaged off feeders and cultured in ESC medium with 2 µg/ml Dox. Twenty-four hours after passage, cells were culture in N2B27 (without Vitamin A) media without LIF or serum for the duration of the experiment [137]. Gene expression for differentiation markers was assayed by quantitative Real-Time PCR at 24-hour intervals. For immunostaining, cells were fixed with 4% paraformaldehyde in PBS and stained with anti-Nestin (Developmental Hybridoma Bank) and DAPI.

### Quantitative real-time PCR

Trizol-isolated RNA from three biologically independent samples was purified, DNAse treated (DNA free RNA Kit, Zymo Research) and reverse transcribed using a First Strand Synthesis Kit (Invtirogen). cDNA was analyzed in triplicate for each biological sample by quantitative PCR using an ABI Prism 7000 (Applied Biosystems) with Platinum SYBR green qPCR SuperMix-UDG with ROX (Invitrogen). All primers used in this study are listed in Table S11. Data were extracted from the linear range, and the standard curve method was used to obtain relative expression values. Technical replicates were averaged and then biological replicates were averaged. Statistical significance was determined using Graphpad Prism to perform an ANOVA with Bonferroni Correction for multiple testing.

### Definition of SOX2-BRN2-bound H3K4me1 and H3K27Ac Regions in TetO-Brn2 cells

Regions of SOX2-BRN2 co-occupancy in TetO-Brn2 cells were defined as above. To define regions of differential chromatin state between TetO-Brn2 and control cells, we first compared H3K4me1 enrichment in these cells to define regions common to both cell types or unique to one or the other. Common regions were merged and treated as one enhancer if detected in both cell types. A similar analysis was performed for H3K27Ac enrichment. SOX2-BRN2 regions were then compared to regions of H3K4me1 and H3K27Ac in order to define SOX2-BRN2 binding events that resulted in changes in chromatin state between TetO-Brn2 cells and controls.

### Identification of DNA binding motifs within SOX2-bound regions

100 base pair windows around the max peak of SOX2-bound regions (in ESCs and NPCs) regions were analyzed for the presence of overrepresented DNA binding motifs. Similarly, 150 base pair windows around the midpoints between the max peaks of SOX2 and OCT4 or BRN2 (in ESCs or NPCs, respectively) in co-bound regions were analyzed for the presence of overrepresented DNA binding motifs. We used a hypothesis-based approach to identify known protein-DNA recognition elements enriched in each dataset. The set of hypotheses are derived from all vertebrate position-specific scoring matrices (PSSMs) from TRANSFAC [138] filtered for sufficient information content (IC>8 total bits). As many of these motifs are redundant, we clustered them based on pairwise distance by KL-divergence of the PSSMs using Affinity Propagation. The TAMO programming environment [139] was used to store the PSSMs and score sequences. We used two approaches to identify overrepresented motifs. All motifs discussed in the paper were found by both methods except for M00145 in SOX2 bound sites in NPCs which was only found by the first approach described below.

In the first approach, we determined whether motifs were overrepresented in a foreground set of all bound regions (SOX2-bound or SOX2-POU co-bound, depending on the analysis) compared to a background set of randomly generated sequences which matched the GC content of the foreground using the Mann-Whitney-Wilcoxon (MWW) ranked sum test. For each independent motif test, sequences were ranked by the maximum motif score in each sequence (across all *k*-mers in the sequence for a motif of width *k*). This ranked list was used to compute the U statistic for the foreground set from which we computed a p-value and applied a Benjamini-Hochberg multiple hypothesis correction. Because many motifs in the databases are very similar to each other, we present the motif within each cluster with the most significant p-value.

In the second approach, we determined whether motifs were overrepresented in a foreground set of 1,000 randomly selected bound regions compared to a background set of randomly generated sequences matching the GC content of the foreground using THEME [140]. A β value of 0.7 and 5-fold cross-validation (CV) were used as THEME parameters. Statistical significance of the CV-error was calculated using randomization of 25 trials and multiple hypothesis corrected using the Benjamini-Hochberg procedure. As in the MWW tables, we present the motif within each cluster with the most significant p-value.

### Genome-wide distances between SOX2 and cell-type-specific POU factors

Distances between SOX2 bound sites and cofactor bound sites in ESCs and NPCs were calculated as follows. Overlapping regions of SOX2 and POU factors were defined as regions with at least 1-bp of overlap. Peaks from these overlapping regions were then used to define distances between the bound factors. In particular, we calculated distances between SOX2-BRN2 site pairs (NPCs), and SOX2-OCT4 site pairs (ESCs). Site pairs were defined by matching each SOX2 bound site to the closest cofactor bound site within 200 bases. Distance was calculated as the cofactor chromosomal coordinate subtracted from the SOX2 chromosomal coordinate.

### Analysis of spacing between motif matches

Spacing between SOX and OCT sites was determined using a motif-based approach to determine specific spatial arrangement of the motifs in SOX2-OCT4 (ESCs) and SOX2-BRN2 (NPCs) co-bound regions. Max motif scores were calculated as described above and normalized as in Equation 1. Motif matches to SOX were defined as normalized scores greater than 0.85 to a general SOX TRANSFAC matrix, M01308. Similarly, OCT family motif matches were defined as normalized scores greater than 0.85 to a general OCT TRANSFAC matrix, M00342. For each sequence *i* and motif *j*, a motif score *s_ij_*: 
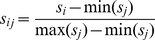
 was calculated. Spacing was defined as the number of base positions between the OCT4 and SOX2 motif matches relative to the SOX2 motif match. OCT-SOX motif pairs were associated with the previously defined “canonical”, “order”, “diverging”, and “converging” orientations [86].

### Gene expression/motif enrichment heat maps

The Mann-Whitney Z-score test result was used to rank all vertebrate TRANSFAC motifs in order of enrichment for SOX2 and OCT4 bound regions in ESCs, and SOX2 and BRN2 bound regions in NPCs. The change in rank (ΔRank) from ESC SOX2-OCT4 bound regions to SOX2-BRN2 bound regions was determined for each motif. Motifs were filtered to include only motifs with a rank less than or equal to 200 in the two ranked lists. Gene expression fold change was determined for each transcription factor associated with at least one TRANSFAC motif. After assigning a pseudocount of 1 to the normalized Affymetrix gene expression values for each transcription factor at the ESC and NPC stages, the log (base 2) of the fold change was calculated. Motifs were mapped to associated transcription factors according to the vertebrate all profile accessible on ExPlain 3.0 containing 656 motifs. The TF with the fold change that best agreed with the ΔRank of the associated motif was chosen as the ‘representative’ factor for the motif (for instance, if ΔRank was negative, the associated transcription factor that had the most negative log-transformed fold change was chosen.) Scaled motif ΔRank values and the associated log-transformed gene expression fold change values were sorted in order of log-transformed gene expression fold change, and viewed in a heatmap (Spotfire, TIBCO). Only motifs that had associated transcription factor expression values were considered.

### Agreement between ΔRank of motif and expression fold-change of associated factor

A spearman correlation coefficient was calculated between the ΔRank values of the motifs and the expression-fold change of their associated transcription factors. This required each motif to be associated with a single transcription factor. In the case where multiple transcription factors are known to bind a single motif, the TF was selected as described in the previous section. The significance of the spearman correlation coefficient was assessed by a Monte-Carlo algorithm. The input to the Monte-Carlo algorithm was a table in which each row of the first column was a motif and each row of the second column was the set of transcription factors known to bind the motif. The column containing the motifs was randomly permuted 100,000 times (thereby randomizing the associations of transcription factors to motifs), and the process of selecting a single transcription factor to be associated with each motif was repeated. After associating each motif with a single random transcription factor, the spearman correlation between the ΔRank of the motifs and the log-fold-change in expression of the transcription factors was computed. The fraction of randomized tables that produced a higher spearman correlation than the original table was reported as the p-value. Only motifs for which the rank in either the SOX2-OCT4 list or the SOX2-BRN2 list was in the top 200 were used. The motifs also had to have at least one associated transcription factor for which gene expression data was available.

### Target gene networks

Genomic intervals corresponding to enriched transcription factor binding motifs in SOX2-BRN2 bound regions were determined. The single nearest gene to a given region was determined using the GREAT algorithm. Genes associated with a motif having a motif similarity score of equal to or greater than 0.85 (439 NF-I motif regions and 251 RFX motif regions) were used to generate a non-redundant target gene list. This gene list was then used as the input for Ingenuity Pathway Analysis. Ingenuity recognized 431 NF-I associated genes and 249 RFX associated genes. Overlap of genomic regions containing motif sequences was performed using Galaxy.

## Supporting Information

Figure S1 Comparison of SOX2 ChIP-Seq datasets in ESCs and NPCs. (A) Correlation between published and replicate ChIP-Seq datasets. (B–D) Overlap of SOX2-bound regions in ESCs (blue) and NPCs (red) (B) All regions; (C) TSS-associated regions within 1 kb of an annotated TSS; (D) Genes associated with TSS-associated regions. If multiple genes were associated with a single SOX2-bound region, all genes were included in this list. Increase in overlap as compared to (C) reflects SOX2 binding at distinct sites within the same promoter in ESCs compared to NPCs.
(EPS)

Figure S2 Overlap of SOX2-bound enhancers reveals SOX2 occupies distinct enhancers of the same gene in ESCs and NPCs. (A) Overlap of enhancer-associated SOX2-bound regions in ESCs and NPCs. (B) Overlap of nearest gene to SOX2-bound enhancers in ESCs and NPCs. Increase in overlap results from distinct enhancer regions mapping to the same gene being occupied by SOX2 in ESCs as compared to NPCs. (C, D) Overlap of genes linked to SOX2-bound promoters and enhancers in ESCs (C) and NPCs (D). By including the genes lined to SOX2-bound enhancers, knowledge of the network controlled by SOX2 increased 3.3 fold (3,113 added genes) and 1.4 fold (2,942 added genes) in ESCs and NPCs, respectively. (E) Pie chart depicting fractions of genes associated with SOX2-bound poised enhancers in ESCs which become associated with SOX2-bound poised enhancers in NPCs (purple), with SOX2-bound active enhancers in NPCs (green), or neither (blue). If a gene gained both a poised and active enhancer, it was grouped with active genes. p-values reflect the probability, based on a cumulative hypergeometric distribution, of obtaining by chance an overlap as high as or higher than that which was observed. (F) Expression of genes linked to SOX2-bound poised enhancers in ESCs (left, blue) and the subsets that become associated with SOX2-bound poised enhancers (center, red) or with SOX2-bound active enhancers (right, red) in NPCs. If a gene gained both a poised and active enhancer, it was grouped with active genes. * reflects p-value<0.0001, unpaired T-test, two tailed. y-axis reflects relative gene expression level within the indicated cell type.
(EPS)

Figure S3 Comparison of SOX2-POU bound sites in ESCs and NPCs. (A) Fraction of total start sites or total marked enhancers associated with BRN2 in NPCs. (B) MEME motif analysis of highest enriched ChIP regions for OCT4 in ESCs and BRN2 in NPCs, demonstrating the similarity in their motif preference. (C) Venn diagram reflecting overlap of bound regions of OCT4 in ESCs compared to BRN2 in NPCs (D–F) Venn diagrams depicting overlap of SOX2 and OCT4 (D) All bound regions; (E) Genes associated with TSS associated regions; (F) H3K4me1 and/or H3K27Ac marked enhancers. (G) Gene plot of the *Oct4 (Pou5f1)* locus, a known SOX2-OCT4 target. y-axis corresponds to reads per million. Genomic positions reflect NCBI Mouse Genome Build 36 (mm8). Gray boxes indicate regions of SOX2-OCT4 co-occupancy. * denotes known enhancer region. (H–I) Venn diagrams depicting overlap of SOX2 and BRN2 in NPCs (H) All bound regions; (I) Genes associated with TSS associated regions; (J) H3K4me1 and/or H3K27Ac marked enhancers. (K) Gene plots of the *Sox2* and *Nestin* loci, which contain known SOX2-BRN2 target enhancers. y-axis corresponds to reads per million. Genomic positions reflect NCBI Mouse Genome Build 36 (mm8). Gray boxes indicate regions of SOX2-BRN2 co-occupancy. * denotes known enhancer region.
(EPS)

Figure S4 Characterization of TetO-Brn2 inducible system. (A) Schematic of transgenic system for inducible expression of *Brn2* from the *Col1A1* locus (adapted from [141]). (B) qRT-PCR time course in control (gray) and *Brn2* induced (black) differentiating ESCs. y-axis represents relative expression normalized to *Gapdh* in 3 biological replicates, measured in triplicate. * denotes p-value<0.05, * denotes p-value<0.01 based on ANOVA with Bonferroni correction. (C) ESC lines were exposed to doxycycline and after 24 hrs. the medium was replaced with N2B27 medium. Cells were harvested after 24 hrs. in N2B27 and represented the day 1 time point. (D) Phase contrast images of control and *Brn2* inducible ESCs at day 2 of differentiation used for ChIP-Seq analyses. (E) Quantification of BRN2 and SOX2 bound regions that map within 1 kb of annotated TSSs and those which are distal to TSSs in TetO-Brn2 cells. (F, G) Metagene analysis within 701 regions depicted in Figure 4C, depicting higher ChIP-Seq density in SOX2 day 2 ChIP compared to SOX2 ESC ChIP (F) and in BRN2 day 2 ChIP compared to OCT4 ESC ChIP (G). y-axis represents WCE-corrected average reads per million in indicated datasets.
(EPS)

Figure S5 Examples of SOX-OCT composite motifs in the four possible configurations. N^n^ between the SOX and OCT motifs reflects the variability of the spacer between these motifs at target loci, where n can be any number greater than or equal to zero. Blue boxed motif represents canonical SOX-OCT motif preferred by SOX2-OCT4 in ESCs when n = 1. Red boxed motif represents SOX-OCT configuration utilized by SOX2-BRN2 more frequently then SOX2-OCT4 when n = 5.
(EPS)

Table S1 ChIP-Seq bound regions for all experiments. ChIP-Seq bound regions called for SOX2 and OCT4 in ESCs, SOX2 and BRN2 in NPCs, and SOX2, BRN2 H3K4me1, and H3K27Ac in TetO-Brn2 and control cells.
(XLSX)

Table S2 Bound Promoters and Enhancers for ChIP-Seq Experiments. Bound promoters, active enhancers, and poised enhancers for SOX2 and OCT4 in ESCs, and SOX2 and BRN2 in NPCs.
(XLSX)

Table S3 GOstat gene ontology analysis of SOX2-bound promoters. Full lists of GOstat gene ontology IDs enriched in SOX2-bound promoters in ESCs and NPCs.
(XLSX)

Table S4 Microarray gene expression data in ESCs and NPCs.
(XLSX)

Table S5 GREAT analysis of SOX2-bound enhancers in ESCs. Full lists of GREAT terms enriched in regions associated with active and poised enhancers occupied by SOX2 in ESCs.
(XLSX)

Table S6 GREAT analysis of SOX2-bound enhancers in NPCs. Full lists of GREAT terms enriched in regions associated with active and poised enhancers occupied by SOX2 in NPCs.
(XLSX)

Table S7 Hypothesis-driven motif search in SOX2-bound regions in NPCs. Mann-Whitney U test and THEME results for TRANSFAC motifs enriched in SOX2 regions in NPCs.
(XLSX)

Table S8 GREAT analysis of SOX2-BRN2-co-bound enhancers in NPCs. Full lists of GREAT terms enriched in regions associated with active and poised enhancers occupied by BRN2 in NPCs and with active and poised enhancers occupied by SOX2-BRN2 in NPCs.
(XLSX)

Table S9 GREAT analysis of BRN2-bound regions common to TetO-Brn2 cells and NPCs, and SOX2-BRN2-co-bound regions in TetO-Brn2 cells. Full lists of GREAT terms enriched in regions bound by BRN2 in both day 2 TetO-Brn2 cells and in NPCs, and regions co-bound by SOX2-BRN2 in day 2 differentiating TetO-Brn2 cells which are not bound by OCT4 or SOX2 in ESCs or SOX2 in control cells.
(XLSX)

Table S10 Hypothesis-driven motif search in SOX2-BRN2-co-bound regions in NPCs. Mann-Whitney U test and THEME results for TRANSFAC motifs enriched in SOX2-BRN2 regions in NPCs.
(XLSX)

Table S11 qRT-PCR primers used to assay for gene expression in TetO-Brn2 and control cells.
(XLSX)
